# Supplementary material for: Pan-cancer proteogenomic investigations identify post-transcriptional kinase targets
Source: Commun Biol. 2021 Sep 22;4:1112. doi: 10.1038/s42003-021-02636-7 (PMC8458405; doi:10.1038/s42003-021-02636-7)
Supplement: Supplementary file 2 — Supplementary Information [file 42003_2021_2636_MOESM2_ESM.pdf]

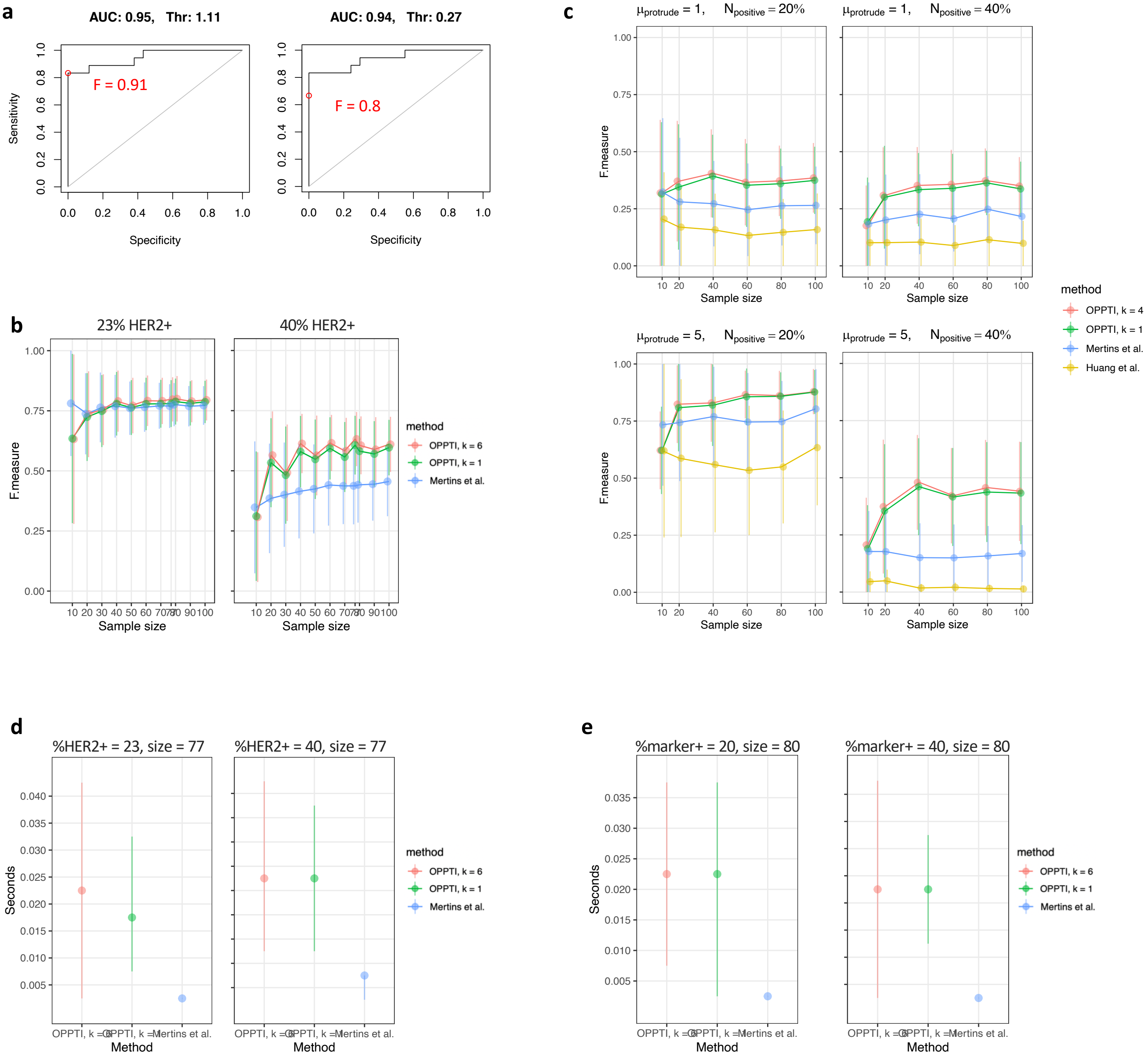

Supplementary Figure 1. Performance benchmarks of the OPPTI algorithm.
(a) Left: OPPTI benchmark in discerning HER2+ samples in CPTAC BRCA retrospective cohort. The performance obtained by default settings is marked by the red circle with corresponding F-measure. Right: benchmark of single-marker approach. The performance obtained by default settings is marked by the red circle with corresponding F-measure. **(b)** Benchmarking methods by balanced undersampling at the true rate (23%) of HER2+ observed in the cohort (left) and by balanced undersampling at a higher rate (40%) of HER2+ (right). Each point represents the mean performance of 100 randomized tests where the whiskers extend by 1 standard deviation. **(c)** Benchmarking methods with synthetic data by balanced sampling at different rates of positives ($N_{positive}$) imposed by protruding expressions at different levels ($\mu_{protrude}$). Each point represents the mean performance of 100 randomized tests where the whiskers extend by 1 standard deviation. **(d)** Running times of the methods for discerning HER2+ samples in CPTAC BRCA retrospective cohort for the specified parameters (%marker = {23, 40}, sample size = 77). Each point represents the mean performance of 1000 randomized tests where the whiskers extend by 1 standard deviation. **(e)** Running times of the methods for discerning a simulated biomarker overexpression for the specified parameters (%marker = {20, 40}, sample size = 80, $\mu_{protrude}$ = 5). Each point represents the mean performance of 1000 randomized tests where the whiskers extend by 1 standard deviation.

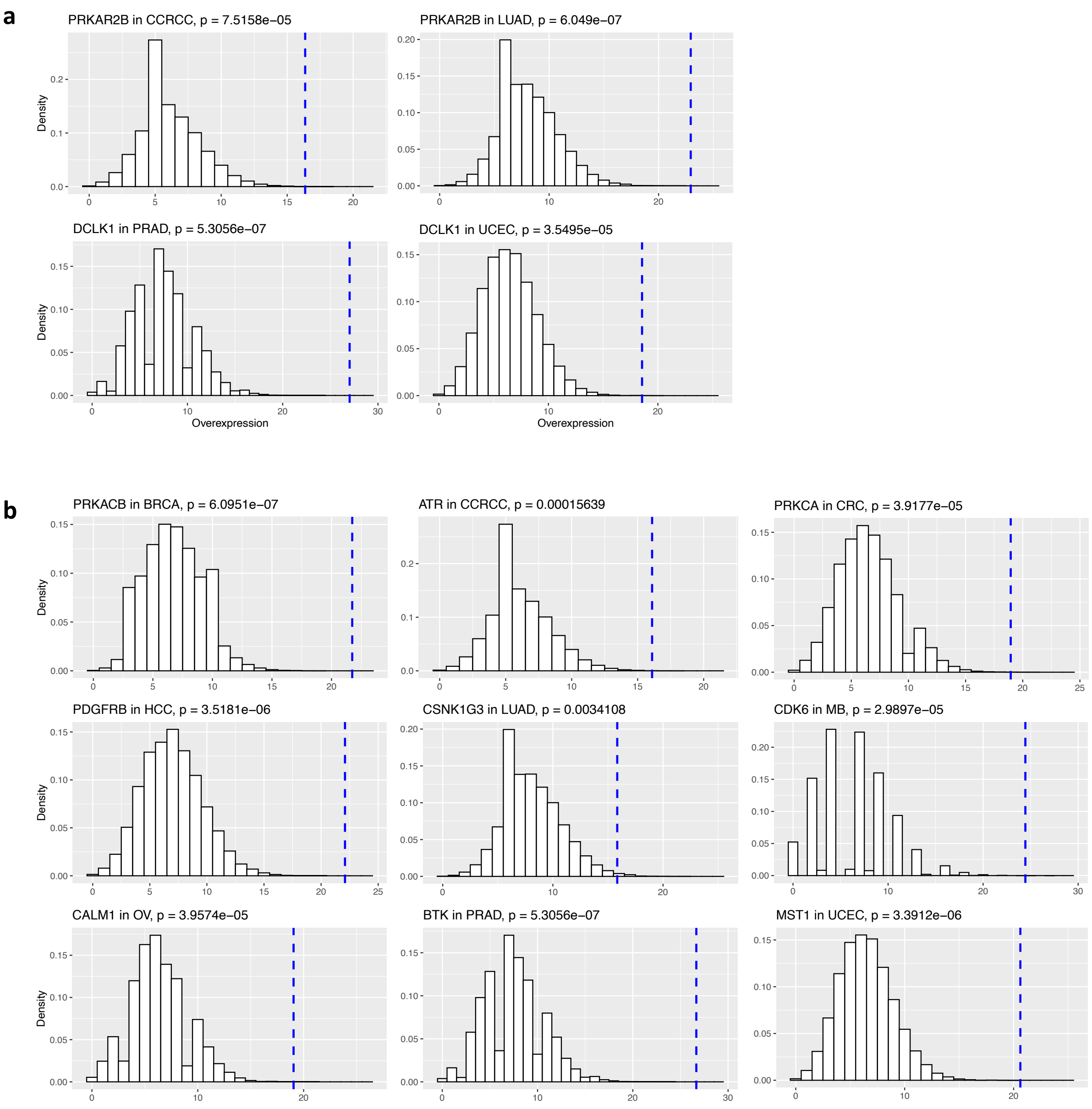

Supplementary Figure 2. Statistical significance of OPPTI overexpressions.
(a) Permutation tests of the druggable kinases with pan-cancer overexpression in **panel c** of **Figure 3**; *p* indicates "p-value". **(b)** Permutation tests of the druggable kinases with cancer-specific overexpression pattern in **panel e** of **Figure 3**.

a

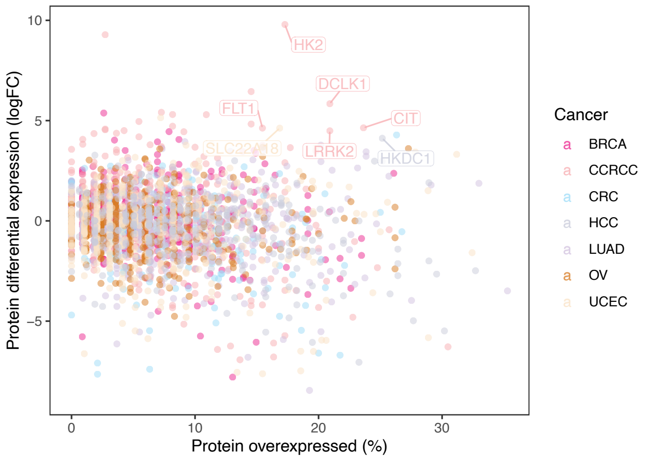

c

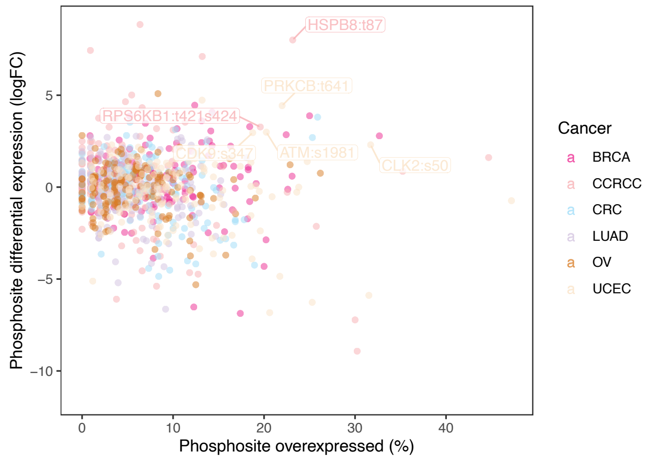

b

Kinase Protein	OverExpressed	Not OverExpressed
DiffExpressed	49	309
Not DiffExpressed	246	1968

d

Kinase Phosphosite	OverExpressed	Not OverExpressed
DiffExpressed	17	100
Not DiffExpressed	75	722

Supplementary Figure 3. Comparison of OPPTI overexpression and DE tests. (a) Comparison of the protein overexpression rates calculated by OPPTI (x-axis) with the case-control differential expression values in tumors (y-axis). **(b)** Contingency table showing the overlap between the overexpressed and the differentially-expressed kinases. **(c)** Same analyses in **panel a** based on the phosphosite overexpression. **(d)** Same table in **panel b** based on the phosphosite overexpression.

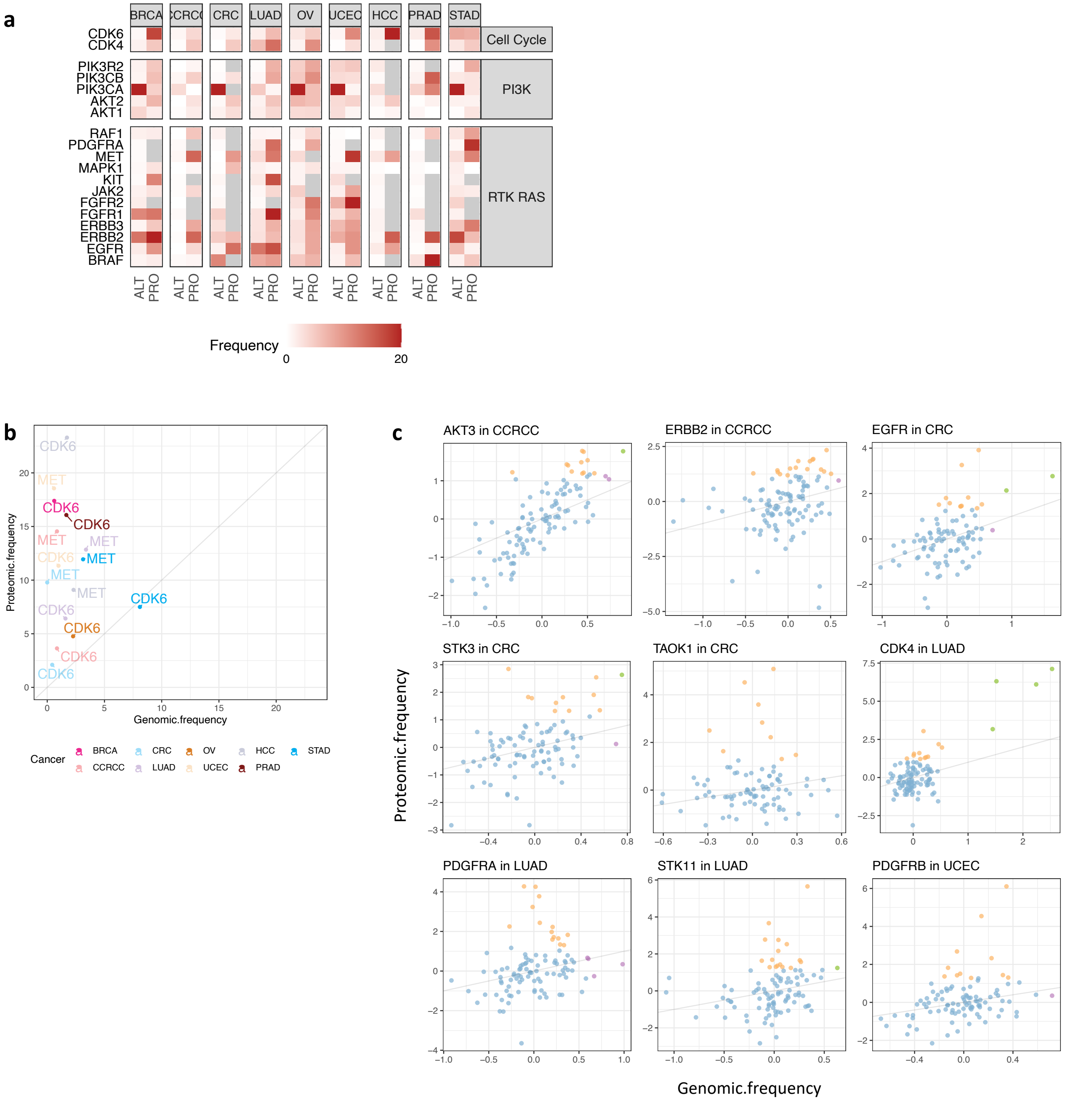

Supplementary Figure 4. Genomic and proteomic alterations of selected kinases.

(a) Genomic and proteomic alteration frequencies of selected kinases. For each cohort, the counts of genomic amplification, mutation and fusion events are unified by regarding multiple alterations in a given site as one, then the alteration rate is computed by these conflated counts with respect to the sample size. **(b)** Proteogenomic alteration patterns in CDK6 and MET kinases. Higher protein-level alterations with respect to genomic-levels are preserved across multiple (≥4) cancer types. **(c)** Kinases with higher protein-level overexpression rates with respect to mRNA-levels (≥3-fold) are labeled.

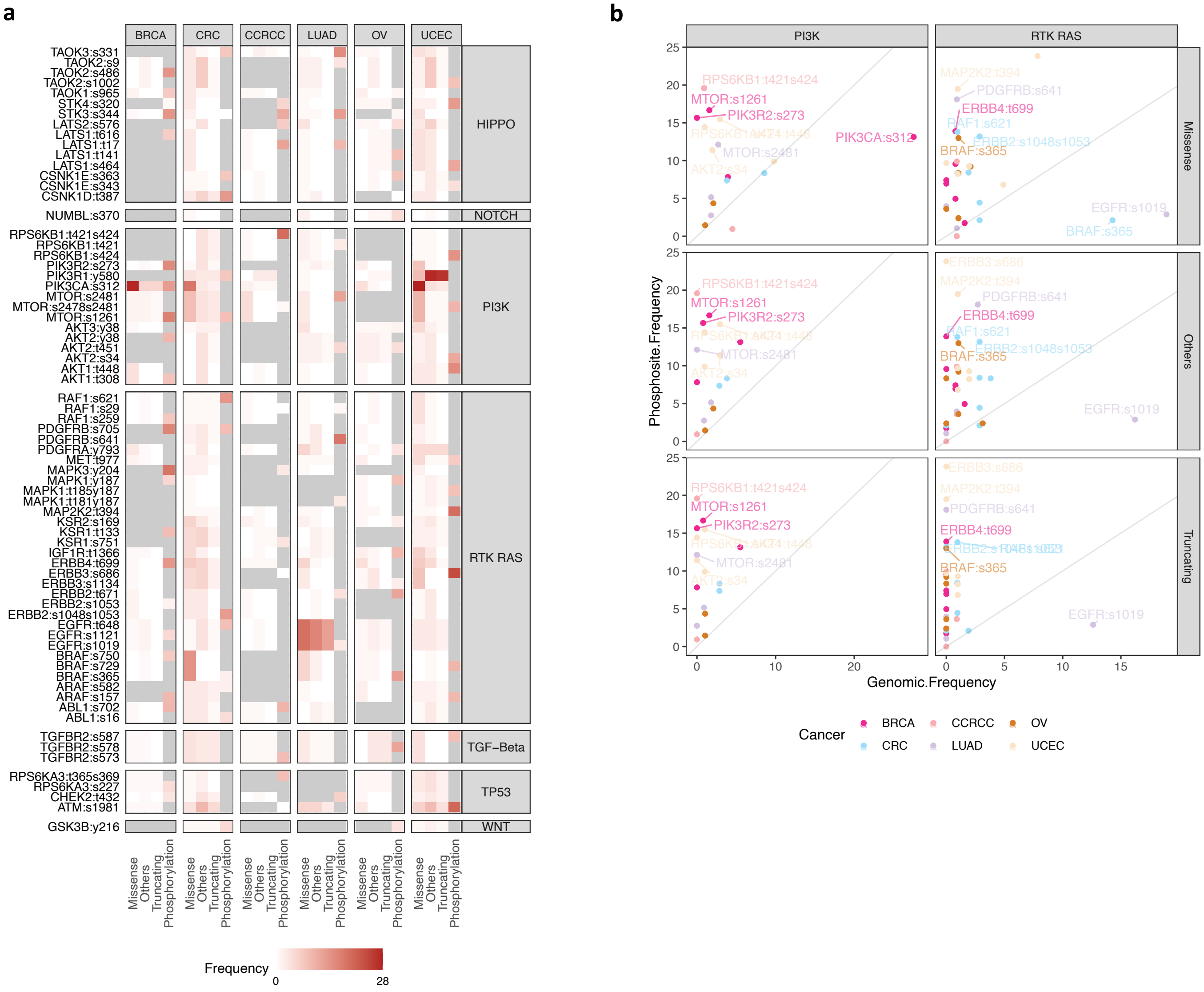

Supplementary Figure 5. Comparison between genomic and phosphosite aberrations. (a) CPTAC genomic alteration and the corresponding phosphosite overexpression frequencies of kinases in the oncogenic signaling pathways. For each cohort, the rates of driver SNVs, (missense and truncating mutations) are individually calculated and displayed with corresponding phosphosite overexpression rate (i.e., the percentage of cases hyperphosphorylated in the cohort). **(b)** The same findings in **panel a** demonstrated in scatter plot.
