# Supplementary material for: Pan-cancer proteogenomic investigations identify post-transcriptional kinase targets
Source: Commun Biol. 2021 Sep 22;4:1112. doi: 10.1038/s42003-021-02636-7 (PMC8458405; doi:10.1038/s42003-021-02636-7)
Supplement: Supplementary file 3 — Description of Supplementary Files [file 42003_2021_2636_MOESM3_ESM.pdf]

Description of Additional Supplementary Files

File name: Supplementary Data

Description: Spreadsheets containing the Supplementary Data 1-9, and the related data used for plotting main figures and supplementary figures.

Supplementary Data 1: Differentially-phosphorylated kinases between tumor and normal samples. The positive fold change (FC) represents hyper-phosphorylation in "tumour" relative to normal.

Supplementary Data 2: Genomic alteration and protein overexpression (the percentage of overexpressed cases in the cohort) frequencies of kinases in the oncogenic signaling pathways.

Supplementary Data 3: Cancer-protein pairs with substantially higher (≥ 3-fold) protein overexpression rate (with P ≥ 10%) than genomic alteration rate.

Supplementary Data 4: Overexpressed kinases with FDA-approved or preclinical drugs in DGIdb that also showed expression-driven cancer dependency (R ≤ -0.3, FDR < 0.05) in cancer cells of the same lineage.

Supplementary Data 5: Novel kinase protein targets that show overexpression in primary patient tumours and expression-driven dependency (R ≤ -0.3, FDR < 0.05).

Supplementary Data 6: Overexpression of kinases that are correlated with up-regulation of their corresponding pathways.

Supplementary Data 7: Hyper-phosphorylation of kinases that are correlated with up-regulation of their corresponding pathways.

Supplementary Data 8: The overview of the proteomics/phosphosite data sets.

Supplementary Data 9: The overview of the mRNA data sets.
